# Failure criterion of silver nanowire electrodes on a polymer substrate for highly flexible devices

**DOI:** 10.1038/srep45903

**Published:** 2017-04-05

**Authors:** Donggyun Kim, Sung-Hoon Kim, Jong Hak Kim, Jae-Chul Lee, Jae-Pyoung Ahn, Sang Woo Kim

**Affiliations:** 1 Clean Energy Research Center, Korea Institute of Science and Technology(KIST), Seoul 02792, Republic of Korea; 2 Department of Chemical and Biomolecular Engineering, Yonsei University, Seoul 03722, Republic of Korea; 3 Advanced Analysis Center, Korea Institute of Science and Technology(KIST), Seoul 02792, Republic of Korea; 4 Department of Materials Science and Engineering, Korea University, Seoul 02841, Republic of Korea; 5 Clean Energy & Chemical Engineering, KIST campus, University of Science and Technology (UST), Seoul 02792, Republic of Korea

## Abstract

Nanomechanical characteristics of standalone silver nanowires (Ag NWs) are a key issue for providing a failure criterion of advanced flexible electrodes that are trending towards smaller radius of curvatures (ROCs). Through *in-situ* tensile and buckling tests of pentagonal Ag NWs, we demonstrated that the intrinsic fracture strain provides a significant criterion to predict the mechanical and electrical failure of Ag NW electrodes under various strain modes, because the decrease in fracture strain limits figure of merit of flexible devices. The Ag NW electrodes on a polymer substrate exhibited a strain-dependent electrical failure owing to the unique deformation characteristics with a size-dependent brittle-to-ductile transition of the five-fold twinned Ag NWs. All the Ag NWs greater than approximately 40 nm in diameter exhibited brittle fracture with a size-independent stress-strain response under tensile and buckling modes, which leads to the electrical failure of flexible electrodes at the almost same threshold ROC. Meanwhile, the higher ductility of Ag NWs less than 40 nm in diameter resulted in much smaller threshold ROCs of the electrodes due to the highly extended fracture strains, which can afford a high degree of freedom for highly flexible devices.

With recent increasing demand for flexible displays (FDs) for mobile electronic devices, researchers have advanced flexible electrodes from curved devices to wearable and foldable devices^1^,^2^,^3^,^4^,^5^. Because these FDs are under various stress states including tensile, compression, and bending^6^,^7^,^8^,^9^, the developers of FDs have used the threshold ROC of FDs as an electrical failure criterion^1^,^6^,^10^,^11^,^12^,^13^. These threshold ROCs, at which the electrical resistance increases over 10%, have been reduced from 10 mm for bendable displays to less than 5–3 mm for rollable and foldable displays, respectively^1^,^13^,^14^,^15^. As the technology trend toward reducing ROCs in futuristic FD’s, we are encountering material challenge that prevents from the electrical and mechanical degradation of flexible electrodes under strain-dependent deformations^9^,^16^,^17^,^18^. At ROCs less than 5 mm, for example, indium tin oxide (ITO) films that are typically used in transparent electrodes go through the degradation process by cracking or channeling defects by bending stress at the crack onset strain (or critical strain) over 0.5%^9^,^17^,^18^, resulting in an abrupt increase in their electrical resistivity^17^,^18^,^19^,^20^,^21^,^22^. This degradation behavior of electrodes below the threshold ROCs makes future FD applications more difficult to develop.

Many researchers have regarded Ag NWs as an alternative transparent electrode material^23^,^24^ because of their high flexibility and good mass productivity via the wet-polyol coating processes^25^, which allow Ag NWs to be used in industrial FD technologies. A recent bending test using Ag NWs-embedded UV curable polymer electrode provides a possibility that can be enhanced up to less than 1 mm in the threshold ROC^24^. Despite the promising performance of Ag NWs, the research on the mechanical and electrical relationship between the bent electrode-on-substrate and Ag NWs themselves has not yet been reported, because the studies on nanomechanics^26^,^27^,^28^,^29^ and flexible devices^11^,^14^,^16^ have been independently performed by researchers in each field. In this work, thus, the failure criterion of Ag NW electrode films was investigated by measuring the inherent mechanical characteristics of standalone Ag NWs and by relating them with the electrical deformation of the electrode films under tensile bending strain.

The substrate surface that is bent by a specific ROC is under two kinds of stress states, which are divided into tensile stress on a convex surface and compressive stress on a concave surface^8^,^9^,^18^. Considering Ag NWs, which is well adhered to the substrate surface, they will endure stress state identical to the bent substrate surface. Therefore, the failure of Ag NWs depends on the amount of deformation in the bent substrate, which is generally determined by the ROC and thickness of the substrate and overlayer^8^,^17^,^18^. The strain (*ε*) is proportional to the substrate thickness and inversely proportional to the ROC, as in Eq. (1)^8^,^17^,^18^.


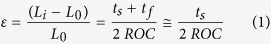


Where *L*_*0*_ and *L*_*i*_ are the initial length and final length before and after loading, *t*_*s*_ the substrate thickness, and *t*_*f*_ the overlayer thickness containing the Ag NWs. Since the overlayer thickness (less than 200 nm) is much less than substrate thicknesses (more than 25 μm) in our study, it can be ignored and the geometrical center of the substrate is also assumed to be the neutral mechanical plane that determines the boundary between tension and compression (see the Supplementary Fig. S1 and Table S1).

In this work, nanomechanical characteristics of Ag NWs are investigated by *in-situ* tensile and buckling tests of the standalone Ag NWs with five-fold twinned boundaries and the results were additionally compared with the electrical properties measured on the electrode films. The fracture origin of the Ag NWs is also investigated by observing the detailed dislocation evolution at deformed regions using transmission electron microscopy (TEM). Based on our experimental results, we discuss the relationship between the intrinsic mechanical properties of the Ag NWs depending on the NW diameter, ROC, and substrate thickness for the highly flexible transparent electrodes, which provides a significant criterion for predicting the mechanical and electrical failure of the Ag NW electrodes.

## Results and Discussion

**Figure 1:**
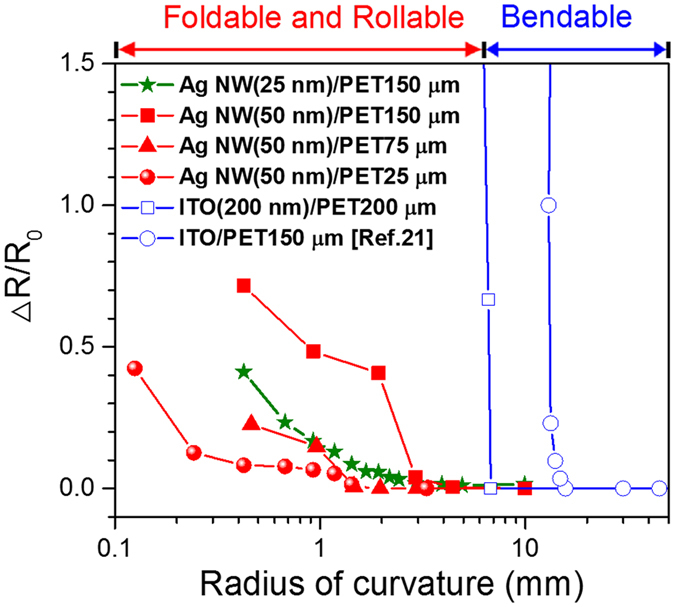
Sheet resistance change (ΔR/R) of the Ag NW electrodes with ROC and a map showing that the fracture of Ag NWs depends on the substrate thickness and ROC.

Figure 1 shows changes in resistance (ΔR/Ro) with ROC during bending tests of transparent conducting films. The Ag NWs and ITO films exhibit a large difference in ΔR/Ro with increasing ROC. The resistances of two ITO films get abruptly worse at the ROCs of 7 and 20 mm, depending on PET thickness. This thickness dependence of the ROC also equally appears in the Ag NW films, whereas those resistances exhibit a sluggish increase even at less than the ROCs of 3 mm, which is the minimum threshold value required in foldable or rollable displays^1^. With decreasing the substrate thickness from 150 to 25 μm at a constant NW diameter of 50 nm, the ROC fracture threshold declines from 3 to 0.3 mm, which is applicable to the highly flexible displays. Likewise, the smaller NWs have resulted in the smaller the ROC fracture threshold owing to the significant decrease in the change of electrical resistance. When the NW’s diameter becomes smaller from 50 to 25 nm at the same substrate thickness of 150 μm, for example, the ROC fracture threshold has gotten much decrease from 3 to 1.3 mm. The change in resistance of the electrodes on substrate under tensile bending strain is closely related to the failure of NWs themselves, indicating that it stands in need of understanding nanomechanical properties of standalone NWs. The failure behavior under bending-mode deformations^18^ can be determined by the various factors of Ag NWs such as orientation, diameter, length, concentration, adhesion between NWs and substrate, and so on. In this work, therefore, the following assumptions have been made for the bending test of film electrodes on substrate:

1. The Ag NWs on the substrate are randomly aligned to the bending direction. The maximum bending force is applied to the parallel direction of bending and for tilted NWs, the applied bending force can be simplified by a proportional cosine relationship between the NW’s orientation and loading direction.
2. The NWs are immobile on substrate because the adhesion between NWs and substrate is strong enough to resist bending force. The constrain force at the junction of NWs can be ignored due to their small bonding strength.

**Figure 2:**
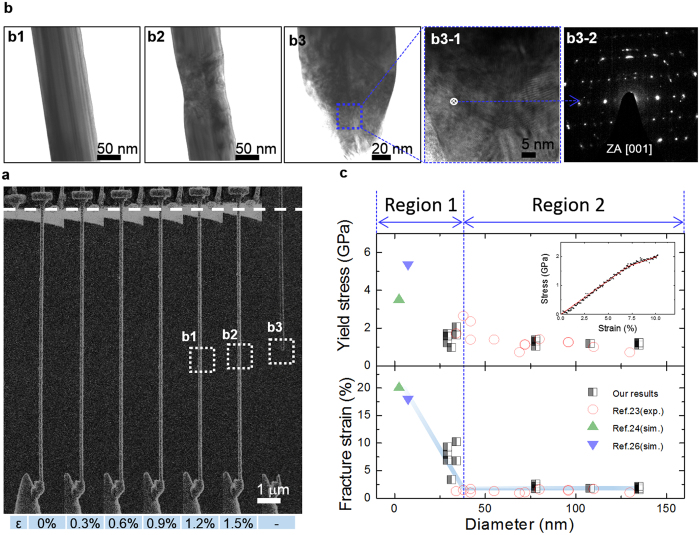
Facture behavior and intrinsic mechanical properties of a five-fold Ag NW. (**a**) SEM snapshots during the tensile deformation of a <110> Ag NW captured from Supplementary Movie S1, showing the entire tensile process of the Ag NW until final fracture. (**b**) TEM images showing fracture behavior of the 5-fold single Ag NW: (b1) Before deformation, (b2) a necking phenomenon at a local region, (b3) a fractographic image of Ag NW and (d3-2) a selected area diffraction (SAD) pattern of a magnified image (b3-1), showing various fragmented spots. (**c**) Yield stress and fracture strain versus the diameter of Ag NWs that have been reported so far, which are comparable to our experimental values with the reported values. In the inset, a strain-stress curve implies that the smaller Ag NW exhibits the higher ductility in Region 1 (Supplementary Table S2).

**Figure 3:**
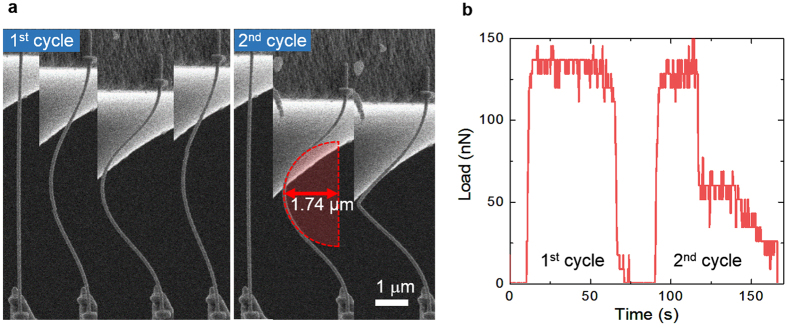
Cyclic compressive loading tests of the five-fold Ag NW and its corresponding load–time curve. (**a**) A series of SEM micrographs showing the cyclic buckling phenomena of an Ag NW under compressive load. In the second cycle, the Ag NW is fractured under a compressive strain that exceeds the fracture threshold of 1.74 μm ROC. (**b**) A load–time curve corresponding to the cyclic compressive loading tests; the curve shows the stress required for buckling.

Under the assumptions, we can ignore the effects of orientation, adhesion, and concentration or length of NWs on the ROC fracture threshold, when the transparent conducting films are bent by a collapsing radius test method. Considering a geometric shape of the substrate in the bending state, the Ag NWs on the convex substrate are under tensile load, vice versa for the concave substrate (see the Supplementary Fig. S1). In order to study the mechanical failure of NW films, the intrinsic mechanical properties of standalone Ag NWs are investigated by *in-situ* buckling (bending) and tensile tests in a dual beam FIB equipped with two nanomanipulators as shown in Figs 2 and 3 (see the Supplementary Fig. S2 for the schematic drawing of bending and tensile tests).

Figure 2 shows SEM and TEM images with mechanical properties of standalone Ag NWs during *in-situ* tensile tests (see the Supplementary Movie S1), showing a representative stress-strain curve in the inset. The Ag NW comprises a pentagonal shape in cross-section area, a growth direction of <110>, the length of approximately 12 μm, and the diameter of 135 nm (see the Supplementary Fig. S3 and Table S2). The TEM images are also captured with tensile strains identical to the SEM cases. According to nanomechanics in single-crystalline FCC nanowires^30^,^31^,^32^,^33^, a twin nucleates after the nucleation of a partial dislocation at a slip plane with high Schmid factors and propagates along a loading direction. This propagation induces a uniform and long-range deformation of the FCC NWs without concentrating stresses by defects or obstacles pre-existing in NWs. In contrast, the Ag NWs with five-fold twin boundaries reveal a large difference in mechanical behaviors. They are brittlely fractured as shown in the inset of Fig. 2(c). The TEM results show the complicated dislocation structures by lots of stacking faults and tangled dislocations at necking and fracture regions, resulting in a polycrystalline-like selected-area diffraction (SAD) pattern. In the previous report^27^, internal defects such as five-fold twin boundaries are known to inhibit the long-range propagation of stacking faults or dislocations. The necking phenomenon in Ag NWs easily leads to the brittle fracture rather than uniform deformation that has been observed in the defect-free other FCC NWs.

On the other hand, Fig. 2c shows a size-dependent brittle-to-ductile transition of Ag NWs, which is separated by Region 1 and 2. In the Region 1 meaning below the diameter of 40 nm, the mechanical properties are variable with the diameter of NWs in the range of the yield strength of 1.22–5.69 GPa, which is approximately 20–100 times higher than a yield stress of 54 MPa in bulk Ag. This size dependence was also verified from the fracture strain. The previous simulation results^27^,^28^,^29^ have indicated that the pentagonal Ag NWs with diameters less than 7.5 nm have the extremely high tensile fracture strain of ~18%, while the large failure strain at very small diameters has not yet been confirmed by any experiments. In the Fig. 3c, the Ag NWs with diameter of 20–30 nm show fairly large failure strain up to about 18% (see the Supplementary Table S2). Molecular dynamics simulations reveal that the size-dependent strain hardening in the five-fold twinned Ag NWs below the diameter of 15 nm is attributed to the obstruction of surface-nucleated dislocations by twin boundaries^33^.

The Ag NWs that are characterized by brittle fracture in the Region 2 didn’t exhibit the size-dependent fracture strain. Regardless of the size, those mechanical properties are almost constant in about fracture strain and yield stress of 2% and 1.2 GPa, respectively (see the Supplementary Table S2 for more details). The constant mechanical properties are mainly attributed to the 5-fold twin boundaries along the <110> growth direction^26^,^27^,^33^,^34^, because they act as energy barriers of moving dislocations, leading to the brittle failure of Ag NWs. Unlike five-fold twinned Ag NWs, the single-crystalline FCC NWs^30^,^31^,^32^ showed that as the NW size decreases, it is hard to nucleate partial dislocations on the surface and easy to propagate twin via the growth direction of the NW. The behavior of the partial dislocations resulted in the increase of yield strength and fracture strain^31^,^32^. The absence of the large plastic deformation in the Region 2 is closely related to the abrupt change the electrical resistance of flexible electrodes under bending stress. That is, the brittle feature of Ag NWs can trigger an abrupt increase in resistance. Thus, the mechanical results provide a possibility to highly enhance the ROC fracture threshold of FD transparent electrodes by using Ag NWs of small size.

**Figure 4:**
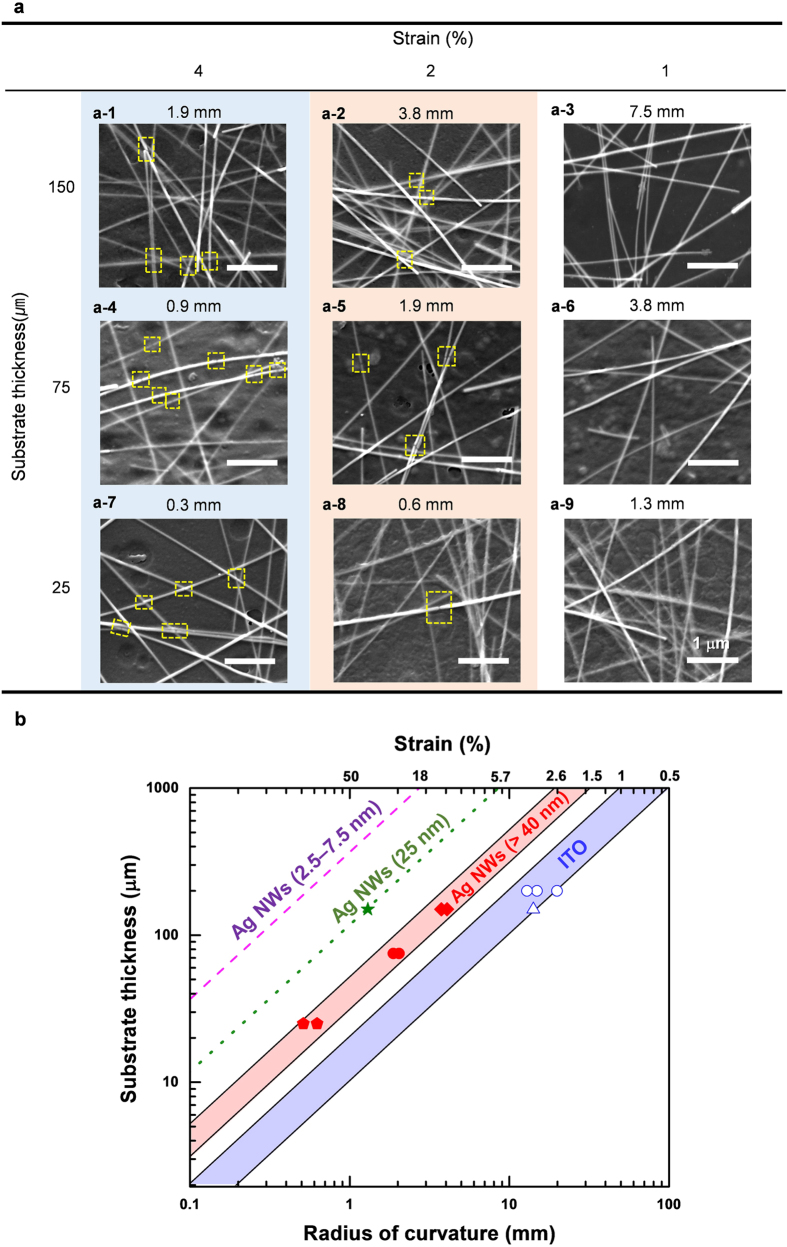
Fracture images and a thickness–ROC map for transparent flexible electrode films under bending. (**a**) SEM images showing brittle Ag NWs with diameters large than 40 nm after bending at various strains under same *ex-situ* condition at the substrate thickness of 150 μm. (**b**) The straight strain lines showing a linear relationship between substrate thickness and ROC correspond to the intrinsic fracture strains of brittle Ag NWs and ITOs. The dashed and dotted lines in the thickness–ROC curve presents the intrinsic fracture strains for ductile Ag NWs with diameters below 7.5 nm^27^,^28^ and 25 nm, respectively.

As shown in the Supplementary Fig. S1, the Ag NWs for FDs can be not only under tensile conditions but also under bending or buckling conditions. Figure 3 shows the well-designed *in-situ* compressive experiment of a Ag NW, a diameter of about 95 nm, by the loading and unloading of a Si cantilever (see the Supplementary Movie S2). The Ag NW is buckled like a bow in the initial compressive stage and fails at a strain of 2.7% by the repeated second loading (see the Supplementary Table S3 and Fig. S4). The buckling load of approximately 136 nN, corresponding to 23.0 MPa, was very low and interestingly was constant during buckling. After failure, the Ag NW endured the compressive load of 50 nN under the dented state. The dotted red semicircle in Fig. 3a indicates the minimum ROC in which the Ag NW can be buckled without failure. In this experiment, the average minimum ROC of the standalone Ag NW was measured by 1.71 μm, which is equivalent to 3.64% engineering strain, and no fracture occurs by the bending (buckling) failure. It means that the Ag NWs under the compressive strain are bearable at the threshold lattice strain nearly twice higher than that by the tensile mode in the Fig. 2. In contrast to the tensile failure in the bending mode, the buckling failure is no longer a significant consideration because the ROC fracture threshold (1.7–2.4 μm in Fig. 3 and Supplementary Table S3) is small enough to use in highly flexible displays. Therefore, the electrical degradation of the Ag NWs transparent electrodes in the Fig. 1 should be evaluated in the convex mode, applying tensile stress to the Ag NWs, like the Fig. 4.

Figure 4a shows the effects of ROC and PET thickness on the fracture of the brittle Ag NWs located on the convex substrate under bending stress (see the Supplementary Fig. S1a and Table S4). The strain is calculated by Eq. (1) depending on the geometric variables of PET thickness and ROC and means the tensile strain applied to the convex surface of electrodes, which is corresponding to the tensile strain applied to Ag NWs on the substrate, according to the previous assumption that the adhesion between NWs and substrate is strong enough. The fracture of Ag NWs begun to occur at the strain over 1.5% because it exceeds the intrinsic fracture strain of standalone Ag NWs (see the Fig. 2c and Supplementary Table S2). Similarly to the Ag NWs, ITO electrodes was also fractured by cracking even at the strain of 1%, which leads to an abrupt increase in sheet resistance (see the Fig. 1 and the Supplementary Fig. S5). These results imply that the intrinsic strain acts as a significant factor to determine the failure of Ag NWs.

Figure 4b provides a map showing the linear relationship between substrate thickness and ROC at different strains of Ag NWs and ITO on the convex substrate. Each strain line in the map expresses the equivalent fracture strains of standalone Ag NWs and ITOs, respectively, and several dots indicate the experimental data plotted as the substrate thickness-ROC curve, showing the 10% increase of electrodes in resistance in the Fig. 1 and the fracture of Ag NWs in the Fig. 4(a). The shaded red and blue areas represent threshold ROCs for Ag NW and ITO electrodes, respectively. For example, the map shows that the Ag NW electrodes consisting of Ag NWs with diameter more than 40 nm electrically fail at an engineering strain between 1.5% and 2.6%, and the ITO electrode films fail at an engineering strain of 0.5–1%. Interestingly, each strain range is well-consistent with the intrinsic yield strain of the brittle Ag NWs and ITO films, respectively, which is evidenced by the experimental data marked by red and blue dots within the transition region of the red and blue area. It reveals that the electrical failure of electrodes at various ROCs and thicknesses of substrate is crucially dependent on the intrinsic fracture strain of electrode materials.

On the other hand, the substrate thickness–ROC map represents that the Ag NWs less than 40 nm in diameter can endure much lower threshold ROC, which is due to the size-dependent brittle-to-ductile transition of Ag NWs as shown in Fig. 2c. With decreasing the NW diameter from 50 to 7.5 nm at a constant substrate thickness of 150 μm, the ROC fracture threshold declines from 3 to 0.4 mm, because the fracture strain of standalone Ag NWs is extended from 2% to more than 18%^27^,^28^, as plotted by the dashed orange line in Fig. 4b. In this respect, the thinner Ag NWs provide a high structural tolerance for the FD applications due to the higher intrinsic fracture strain than thicker Ag NWs.

From the nanomechanical and electrical characteristics of a five-fold Ag NW, we suggested a failure criterion that can evaluate the conductive electrodes of FDs under various stress modes. The Ag NWs with diameters greater than 40 nm in buckling mode exhibited cyclic buckling characteristics without failure up to the threshold ROC of 1.1–2.4 μm, corresponding to the engineering strain of 2.7–5.2% higher than tensile mode. In the *in-situ* tensile test, in contrast, the Ag NWs exhibited brittle fracture by localized necking at the lattice strain of ~2.6%, leading to the abrupt increase of electrical resistance in flexible electrodes. The smaller Ag NWs in diameter, the higher ductility and the lower threshold ROCs. Conclusively, we proved that the ROC fracture threshold as a failure criterion of conductive electrode films is intrinsically determined by the lattice strain of Ag NWs, inducing brittle fracture, that is changed by the size of NWs. The relationship of the mechanical behavior of Ag NWs and the electrical properties of electrodes will provide a high structural tolerance for designing FD applications

## Materials and Methods

### Synthesis of Ag NWs

Ag NWs were synthesized via a modified polyol process^25^. In the first step, 40 mL of ethylene glycol (EG; 99.5%, Daejung Chem. & Metals), which served as a solvent and reducing agent, was added to a 100-mL, three-neck, round-bottom flask in a heating mantle set at 160 °C. Copper(II) chloride dehydrate (CuCl_2_·2H_2_O; 97%, Daejung Chem. & Metals) was added to the flask to a concentration of 0.125 mM CuCl_2_, and the solution was stirred at 160 °C for 1 h. Poly(vinylpyrrolidone) (PVP, Mw = 55,000, Sigma-Aldrich, 1.7 g) as a capping agent was subsequently added to the solution. In the second step, 40 mL of 0.1 M and 40 mL of 0.3 M silver nitrate (AgNO_3_; 99.95%, Next Chimica) dissolved in EG were prepared, respectively. The 40 mL of 0.1 M or 0.3 M AgNO_3_ solution was poured into the CuCl_2_ solution within a short time period of 1 min or less. The mixed solution was maintained at 160 °C for 2 h under magnetic stirring at 300 rpm. After synthesis, the precipitate-containing solution was cooled to room temperature and diluted with 80 mL of ethanol. The diluted solution was centrifuged twice at 3,000 rpm, and the Ag NWs were subsequently redispersed into ethanol.

SEM images of Ag NWs prepared using the aforementioned procedure are shown in Supplementary Figure S3a; NWs are 5–20 μm long and 50–150 nm wide, and in Supplementary Figure S3c; NWs are 5–10 μm long and 20–30 nm wide, and form pentagonal sections. In TEM images in Supplementary Figure S3b and Sd show that the synthesized Ag NWs grew along the <110> direction and had 5 twin boundaries inside the pentagonal sections. For the synthesized Ag NWs, *in-situ* tensile tests were conducted using a dual-beam focused ion beam (FIB, Quanta 3D FEG, FEI), and the crystalline structure of synthesized Ag NWs was analyzed by TEM (Tecnai F20 G2, FEI).

### *In-situ* mechanical test of standalone Ag NWs

The mechanical tests of standalone Ag NWs were performed by tensile and bending tests^26^,^35^ in a dual-beam FIB equipped with two nanomanipulators (MM3A, Kleindiek), including a cantilever-based force measurement system (FMS) that can be used for nanoscale manipulation. The Ag NWs used in the mechanical tests were 24–135 nm in diameter and 0.72–2.65 um in length. An individual Ag NW dispersed on a Si wafer was picked up by the W-tip and welded onto the FMS cantilever by Pt deposition (Fig. 2a and Supplementary Figure S2). The tensile and bending samples were prepared under the same conditions and tested at speeds of 0.12~4.57 nm/s, corresponding to *ε* = 3.9 × 10^−5^ s^−1^–3.9 × 10^−3^ s^−1^, respectively. Movies (Supplementary Movies S1 and S2) were simultaneously recorded with the change of load, where the time is equal to the variation of strain and the load can be converted into the stress by dividing by the area of the nanowire.

### Bending tests of transparent conducting films

Ag NW films were coated by Meyer rod onto poly(ethylene terephthalate) (PET) films with thicknesses of 25, 75, and 150 μm. For the Ag NW films (100 nm thickness, 12 Ω/◽) and ITO films (200 nm thickness, 15 Ω/◽, Peccell Tech., Inc.) sputtered onto Poly(ethylene 2,6-naphthalate) (PEN) substrates with a thickness of 200 μm, bending tests were performed using the collapsing radius test method^16^, as shown in Supplementary Figures S1a and S5a. The ROC of the conducting films was measured by changing the distance between walls (2*R*). After the bending tests, the sheet resistances of the bent samples were measured using the four-point probe method and the images of the fractured Ag NW films were observed by field emission scanning electron microscopy (INSFECT F50, FEI).

## Additional Information

**How to cite this article**: Kim, D. *et al*. Failure criterion of silver nanowire electrodes on a polymer substrate for highly flexible devices. *Sci. Rep.* **7**, 45903; doi: 10.1038/srep45903 (2017).

**Publisher's note:** Springer Nature remains neutral with regard to jurisdictional claims in published maps and institutional affiliations.

## Supplementary Material

Supplementary Figures and Tables

Supplementary Movie 1

Supplementary Movie 2
