# Supplementary material for: Failure criterion of silver nanowire electrodes on a polymer substrate for highly flexible devices
Source: Sci Rep. 2017 Apr 5;7:45903. doi: 10.1038/srep45903 (PMC5381103; doi:10.1038/srep45903)
Supplement: Supplementary Figures and Tables [file srep45903-s1.pdf]

Failure criterion of silver nanowire electrodes on a polymer substrate for highly flexible devices

Donggyun Kim[1,2], Sunghoon Kim[3,4], Jong Hak Kim[2], Jae-chul Lee[4], Jae-Pyoung Ahn[3,★], and Sang Woo Kim[1,5,★]

*These authors contributed equally
**correspondence to: jpahn@kist.re.kr, swkim@kist.re.kr

This PDF file includes:
Supplementary Text
Supplementary Figures S1 to S5
Supplementary Tables S1 to S4
Captions to Supplementary Movies 1 to 2

Other Supplementary Materials for this manuscript include:
Supplementary Movies 1 to 2

List of Supplementary Figures, Tables, and Movies

Supplementary Fig. S1 | Schematic diagrams showing the stresses developed in the electrode materials on the substrate with curvature and the measurement setup for bending test.
Supplementary Fig. S2. Schematic diagrams of in-situ buckling (bending) and tensile test in a dual beam FIB equipped with two nanomanipulators.
Supplementary Fig. S3 | SEM and TEM images of 5-fold Ag nanowires synthesized by modified polyol process.
Supplementary Fig. S4 | Schematic views for the buckling (bending) test of a standalone Ag NW.
Supplementary Fig. S5 | Photograph of an in situ bending test setup and SEM images of ITO films fractured under in situ and ex situ bending stresses.

Supplementary Table S1 | The error showing the difference between neutral mechanical plane and geometrical center
Supplementary Table S2 | Mechanical properties of standalone Ag NWs obtained by tensile tests and by molecular dynamics simulations
Supplementary Table S3 | Fracture ROCs and strains of standalone Ag NWs after buckling test.
Supplementary Table S4 | Substrate thicknesses and ROCs of Ag NW conducting films at constant strains under tensile bending.

Supplementary Movie 1 | Tensile-test movie for an Ag NW.
Supplementary Movie 2 | Buckling (bending) test movie for an Ag NW.

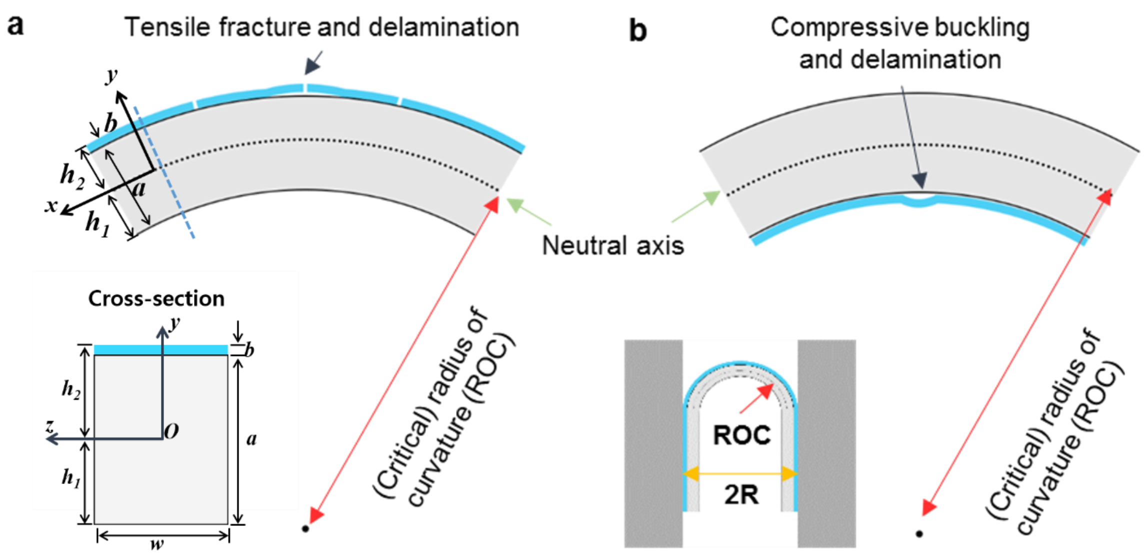

Supplementary Fig. S1. Schematic diagrams showing the stresses developed in the electrode materials on the substrate with curvature and the measurement setup for bending test. (a) The electrode materials under tensile bending stress. In this case, the electrode materials are not under compressive stress. (b) The electrode materials under compressive buckling stress. No longitudinal stresses or strains are present at the neutral axis. The test is performed by moving both plates until the substrate satisfies the designed specific ROC. The electrode materials are considered to be under either tensile or compressive stress, which is different from the standalone NW bending test. The strain induced by the displacement is calculated as follows[1,2]:

$$\varepsilon = \frac{(L_i - L_0)}{L_0} = \frac{\Delta L}{L_0} = \frac{(R - r_n)\theta}{r_n\theta} = \frac{[R - (R - \frac{t}{2})]}{\left(R - \frac{t}{2}\right)} = \frac{t}{2[R - \frac{t}{2}]} = \frac{t}{2[r + \frac{t}{2}]} = \frac{t}{2\,ROC}$$

where ε is the strain, L_i is the final length, L_0 is the initial length, θ is the bend angle, R is the radius of the outermost film, r_n is the length of the neutral axis, r is the radius of the innermost film, t is the thickness, and ROC is the radius of curvature.

As the Ag NWs are too thinner than the PET substrate that we have used in our study, we assumed the geometrical center of PET as the neutral mechanical plane. However, the neutral axis is not always same with the geometrical center in a double layer composite having highly different Young's moduli. Therefore, the error that induced by the difference between geometrical center and neutral axis needs to be calculated and be conformed as follows. The neutral mechanical plane is calculated by following Eqs. (s1) to (s3)[3], based on the stress relationship between two layers.

$$\int_{PET} \sigma_{PET} dA + \int_{Ag} \sigma_{Ag} dA = 0 \tag{s1}$$

$$E_{PET} \int_{PET} y_{PET} dA + E_{Ag} \int_{Ag} y_{Ag} dA = awE_{PET}\left(h_1 - \frac{a}{2}\right) + bwE_{Ag}\left(a + \frac{b}{2} - h_1\right) = 0 \tag{s2}$$

$$h_1 = \frac{\frac{a^2}{2} E_{PET} + abE_{Ag} + \frac{b^2}{2} E_{Ag}}{aE_{PET} + bE_{Ag}} \tag{s3}$$

Here, σ is the normal stress acting on z-y plane at y, y is the distance from neutral axis along y axis, E is the Young's modulus, a and b are the thickness of Ag NW and PET respectively, h_1 and h_2 are the thickness from neutral axis to the upside and downside, w is the width of layer along z axis. The Eq. (s3) indicates the practical neutral mechanical plane calculated by considering the Young's moduli of two layers (E_{PET}= 4 GPa, E_{Ag}=22 GPa). From the Eq. (3), the difference between neutral mechanical plane and geometrical center is represented in Table S1, but the error is too low and the difference between neutral mechanical plane and geometrical center can be ignored. Thus, we assumed that the neutral mechanical plane is nearly the same as the geometrical center in Supplementary Fig. S1.

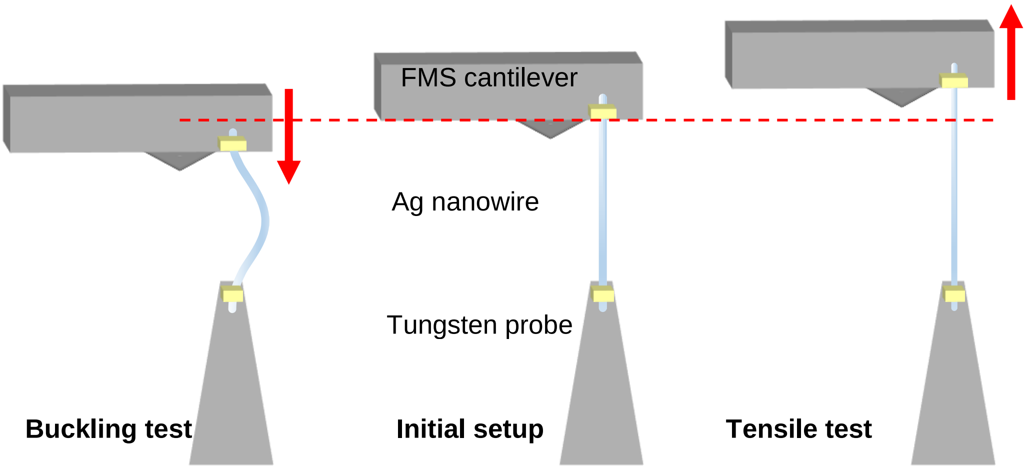

Supplementary Fig. S2. Schematic diagrams of in-situ buckling (bending) and tensile test in a dual beam FIB equipped with two nanomanipulators. A FMS cantilever and a w-tip are used for measuring the mechanical force and pinning the opposite side of a single Ag NW. The in-situ tensile tests and bending tests are carried out by the movement of FMS cantilever.

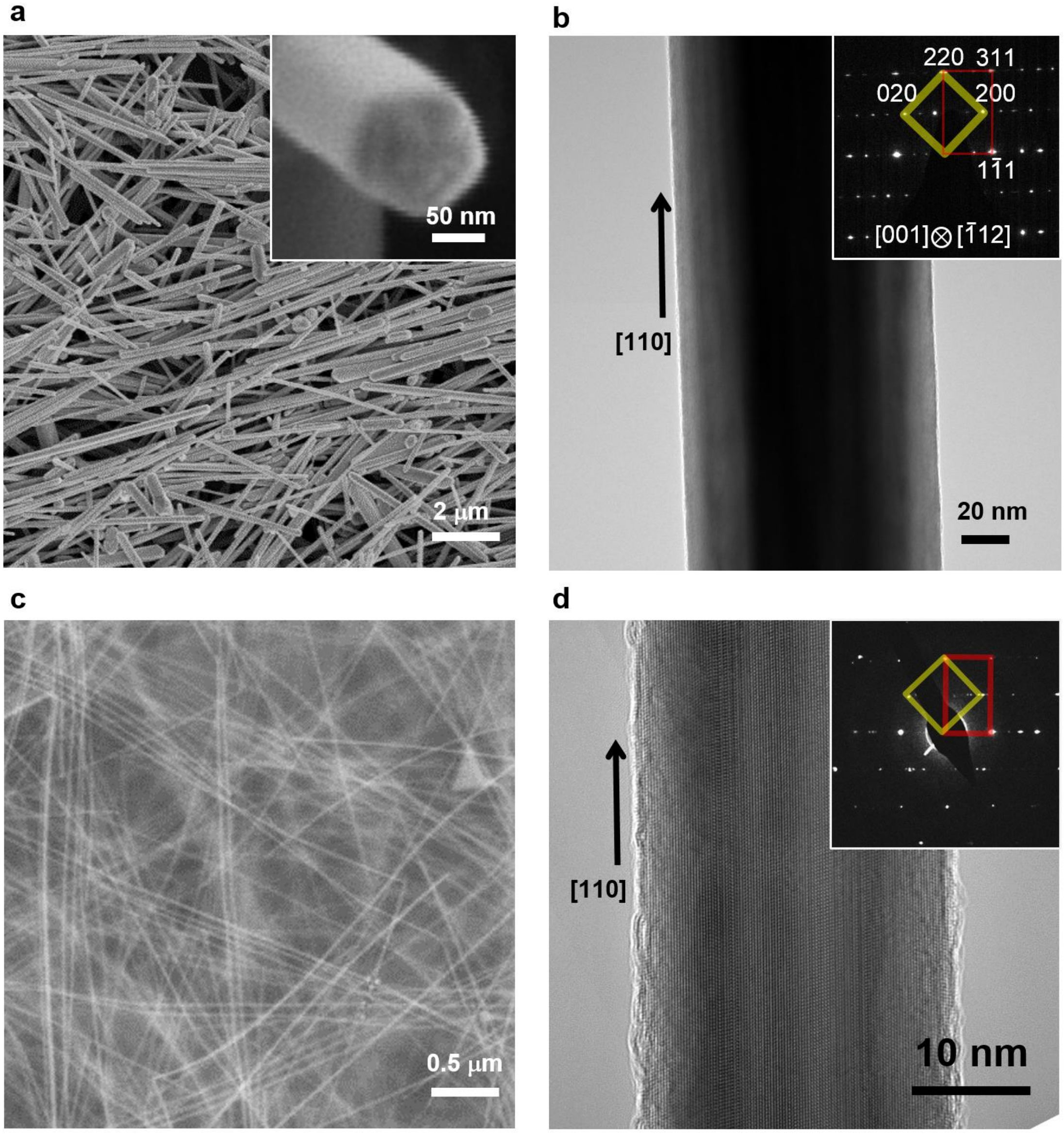

Supplementary Fig. S3. SEM and TEM images of 5-fold Ag nanowires synthesized by polyol process. (a) SEM images of Ag NWs. Those are in the range of 5–10 μm in length and 80–100 nm in diameter. The cross-sectioned SEM image in the inset clearly shows an Ag NW of 5 folded shape. (b) A TEM image and a SAD pattern of an Ag NW (a). In the inset, the SADP indicates the overlapped zone axis of [001] and [112], generally implying that the Ag nanowire consists of 5 twin boundaries and a growth direction of [110]. (c) SEM images of Ag NWs with the range of 20–30 nm in diameter. (d) A TEM image and a SAD pattern of an Ag NW (c).

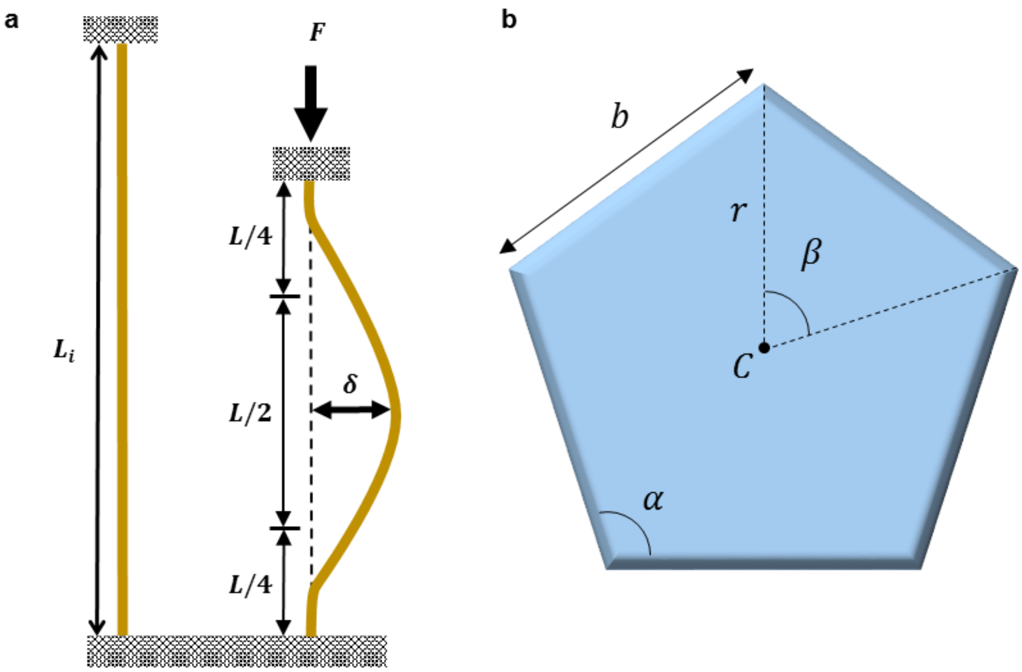

Supplementary Fig. S4. Schematic views for the buckling (bending) test of a standalone Ag NW. (a) Schematic of the nanowire buckling test with two fixed ends. Here, the real bending part is one-half the center area of the whole nanowire length ($L/2$). (b) Cross-sectional view of an Ag NW with a pentagonal shape.

The Young's modulus of a nanowire E is calculated at a critical buckling load F by the following equation[4,5]:

$$\mathrm{F} = \frac{4\pi^2 EI}{L^2}$$

where the moment of inertia of the polygon-shaped nanowire I is given as follows.

$$I_c = \frac{nb^4}{192}(cot\frac{\beta}{2})(3cot^2\frac{\beta}{2} + 1)$$

Here n = 5 and β = 72° are given for the pentagonal shape,

$$I_{c,pentagon} = \frac{5b^4}{192}(\cot 36°)(3cot^2 36° + 1) = 0.240\ b^4$$

where L is the length of a nanowire, δ is the distance from the initial axis, b is the side length of the nanowire, r is the radius of the nanowire, C is the center of the pentagonal cross-section, and β is the central angle for a side. The average Young's modulus was 82 GPa, and the average yield strength was 24.9 GPa.

Finally, the young's modulus measured from bending test is calculated as follows:

$$E = \frac{L^2F}{4\pi^2 I} = \frac{L^2F}{0.960\pi^2 a^4}$$

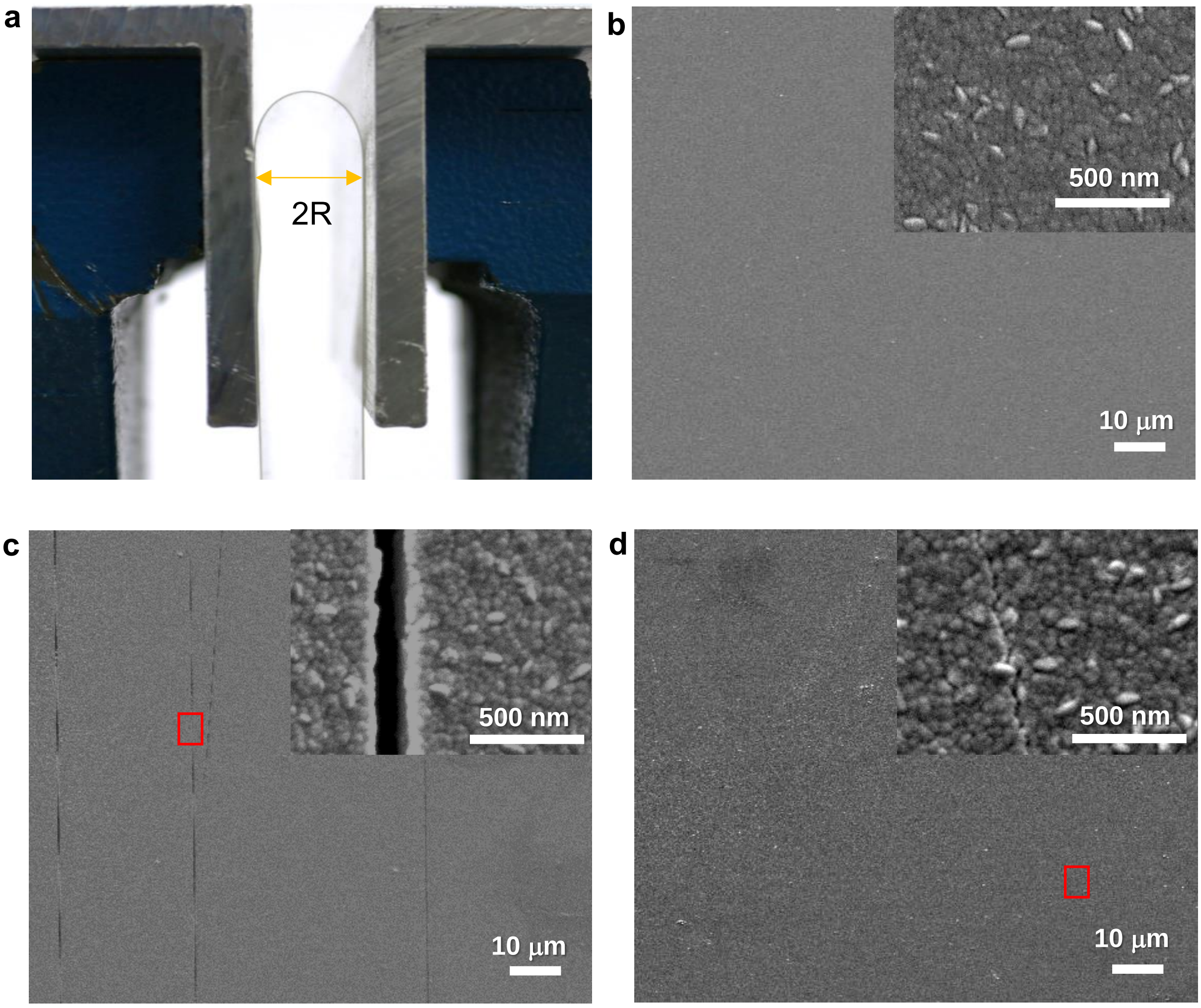

Supplementary Fig. S5. Photograph of an in situ bending test setup and SEM images of ITO films fractured under in situ and ex situ bending stresses. (a) Optical image of a collapsing radius test (ROC = 10 mm, strain 1%). (b) SEM images of ITO before bending. (c) SEM images showing ITO fractured by channeling during in situ bending at 1% strain. (d) SEM images showing self-healed ITO, which is observed after ex situ bending at 1% strain.

Supplementary Table S1. The error showing the difference between neutral mechanical plane and geometrical center

(Unit: %)

Ag (nm) / PET (μm)	30	50	100	150	200	250
25	0.54	0.89	1.75	2.60	3.42	4.22
75	0.18	0.30	0.59	0.89	1.18	1.47
150	0.09	0.15	0.30	0.45	1.47	0.74

Supplementary Table S2. Mechanical properties of standalone Ag NWs obtained by tensile tests (Figure 2 and Supplementary Fig. S2) and by molecular dynamics simulations

Diameter (nm)	Length (μm)	Young's modulus (GPa)	Fracture strain (%)	Yield strength (GPa)	Source
24.4	0.82	37.2	15.03	5.69	Our results
26.9	0.97	32.4	7.71	2.52	
26.9	0.72	54.5	10.52	5.61	
29.2	1.56	42.9	4.70	1.22	
29.2	0.72	43.9	6.55	1.71	
29.4	2.05	52.3	6.65	2.79	
78.0	7.46	60.90	2.13	1.28	
78.0	6.00	54.90	2.55	1.38	
78.0	8.37	62.78	1.68	1.04	
108.0	2.96	68.00	1.75	1.18	
135.0	12.65	62.38	1.96	1.22	
135.0	12.05	71.60	1.53	1.09	
135.0	4.24	73.53	1.67	1.22	
135.0	2.98	57.80	2.11	1.22	
80.0	4.67	94.00	1.55	1.41	Ref. 11 (exp.)
96.0	2.11	86.00	1.47	1.26	Ref. 11 (exp.)
96.0	2.69	94.00	1.35	1.24	Ref. 11 (exp.)
2.6	-	70.00	20.0	3.50	Ref. 12 (sim.)
7.5	-	113.00	18.0	5.37	Ref. 14 (sim.)

Supplementary Table S3. Fracture ROCs and strains of standalone Ag NWs after buckling test. Here, the strains are the tensile strains applied in the outmost side of the Ag NWs under the buckling (Figure 3, Supplementary Fig. S3 and S5a)

Length of Ag NWs (μm)	Diameter of Ag NWs (nm)	Threshold ROC (μm)	Buckling strain (%)
8.11	95	1.74	2.73
6.34	111.7	1.40	3.99
6.45	111.7	1.08	5.17
11.48	134.6	1.93	3.49
13.19	133.2	2.38	2.80
Average		1.71	3.64

Supplementary Table S4. Substrate thicknesses and ROCs of Ag NW conducting films at constant strains under tensile bending (Figure 4a and Supplementary Fig. S4a)

Strain (%)	Substrate thickness (μm)	ROC (mm)
4	150	1.88
4	75	0.94
4	25	0.31
2	150	3.75
2	75	1.88
2	25	0.63
1	150	7.50
1	75	3.75
1	25	1.25

Supplementary Movie 1 | Tensile-test movie for an Ag NW.

The movie shows the entire tensile process of a <110> Ag NW until final fracture.

Supplementary Movie 2 | Buckling (bending) test movie for an Ag NW.

The movie shows the cyclic buckling phenomena of an Ag NW under compressive load. In the second cycle, the Ag NW is fractured under a compressive strain that exceeds the fracture threshold of 1.74 µm ROC.

Supplementary References.

1. Chen, Z., Cotterell, B. & Wang, W. The fracture of brittle thin films on compliant substrates in flexible displays. *Engineering Fracture Mechanics* **69**, 597-603 (2002).

2. Khaled, T. Calculation of bend radii from tensile elongation data, Ref. # ANM-112N-03-11, dated 17 September (2003).

3. Gere, J.M. & Goodno, B. J. Mechanics of materials 8th ed. (ed. Adams, R.) Chap. 6, (Cengage Learning, 2013).

4. James, M. Gere, Mechanics of materials 6th edition. Appendix D, (Thomson Learning, Inc., Belmont, 2004)

5. Smith, D. A., Holmberg, V. C. & Korgel, B. A. Flexible germanium nanowires: ideal strength, room temperature plasticity, and bendable semiconductor fabric. *ACS nano* **4**, 2356-2362 (2010).
